# Neddylation pathway is up-regulated in human intrahepatic cholangiocarcinoma and serves as a potential therapeutic target

**DOI:** 10.18632/oncotarget.2309

**Published:** 2014-08-04

**Authors:** Qiang Gao, Guang-Yang Yu, Jie-Yi Shi, Li-Hui Li, Wen-Juan Zhang, Zhi-Chao Wang, Liu-Xiao Yang, Meng Duan, Hu Zhao, Xiao-Ying Wang, Jian Zhou, Shuang-Jian Qiu, Lak Shin Jeong, Li-Jun Jia, Jia Fan

**Affiliations:** ^1^ Liver Cancer Institute, Zhongshan Hospital and Shanghai Medical School, and Key Laboratory of Carcinogenesis and Cancer Invasion (Ministry of Education), Fudan University, Shanghai, P. R. China; ^2^ Cancer Institute, Fudan University Shanghai Cancer Center, and Department of Oncology, Shanghai Medical College, Fudan University, Shanghai, P. R. China; ^3^ Department of Laboratory Medicine, Laboratory of Molecular Biology of Huadong Hospital, Fudan University, Shanghai, P. R. China; ^4^ Institute of Biomedical Sciences, Fudan University, Shanghai, P. R. China; ^5^ College of Pharmacy, Seoul National University, Seoul, Korea

**Keywords:** Intrahepatic cholangiocarcinoma, Neddylation, NEDD8, MLN4924, Cullin-Ring ligase

## Abstract

Therapeutic intervention in neddylation pathway is an emerging area for cancer treatment. Herein, we evaluated the clinical relevance and therapeutic potential of targeting this pathway in intrahepatic cholangiocarcinoma (ICC). Immunohistochemistry of neddylation pathway components in a cohort of 322 cases showed that E1 (NAE1 and UBA3) and E2 (UBC12) enzymes, as well as global NEDD8 conjugation, were upregulated in over 2/3 of human ICC. Notably, NAE1 was identified as an independent prognosticator for postoperative recurrence (P=0.009) and a combination of NEDD8 and NAE1 provided a better power for predicting patient clinical outcomes. *In vitro* treatment with MLN4924, a small-molecule NEDD8-activating enzyme inhibitor, led to a dose-dependent decrease of viability in both established and primary cholangiocarcinoma cell lines. Additionally, MLN4924 exhibited at least additive effect when combined with cisplatin. By blocking cullins neddylation, MLN4924 inactivated Cullin-Ring ligase (CRL) and caused the accumulation of CRL substrates that triggered cell cycle arrest, senescence or apoptosis. Meanwhile, MLN4924 was well-tolerated and significantly inhibited tumor growth in xenograft model of cholangiocarcinoma. Taken together, our findings indicated that upregulated neddylation pathway was involved in ICC progression and interference in this pathway could be a promising target for ICC therapy.

## INTRODUCTION

Intrahepatic cholangiocarcinoma (ICC) is the second most common primary hepatic malignancy after hepatocellular carcinoma, accounting for approximately 10-15% of primary liver cancer [1]. Rising trends in the incidence of ICC have been well-recognized worldwide [1]. Although effective new treatments have increased survival for many other cancers during the past 30 years, treatment strategies and survival for patients with ICC have improved little. For the few patients suitable for curative resection, a dismal 5-year survival of 30% was documented [2]. Still worse, ICC patients respond poorly to aggressive chemotherapy or radiotherapy. Thus, identifying effective treatment strategies to improve outcome is one of the major challenges and future needs for ICC [3].

Cancer cells depend on oncogenic signals that promote cell cycle progression and prevent cell death/senescence that would otherwise result from aberrant stress. This requires the dysregulation of ubiquitin-proteasome pathway that results in permanent activation of pro-tumor signal cascade [4]. Therefore, modulating the ubiquitin-proteasome pathway represents an attractive anticancer strategy. Indeed, a proteasome inhibitor Bortezomib approved for the treatment of multiple myeloma and relapsed mantle cell lymphoma has gained good clinical response [4]. Targeting protein homeostasis has thus become an attractive therapeutic strategy in human cancers. Due to normal cell toxicity resulted from global inhibition of proteasome-mediated protein degradation by Bortezomib, tremendous efforts have been focused on better specificity by targeting the enzymes upstream of the proteasome [5].

The main control points in ubiquitin–proteasome process include families of E3 ubiquitin ligases [6]. Of note, cullin-RING ligases (CRLs) represent the largest multiunit E3 ubiquitin ligase family, which show high selectivity in degradating proteins and are considered as new targets of interest for cancer therapy [5, 7]. Importantly, NEDD8 modification of cullins, the CRLs essential components, is required for the activation of CRLs [8]. The NEDD8-conjugation cascade, called neddylation, is mediated by E1 NEDD8-activating enzyme (NAE1/UBA3), E2 NEDD8-conjugating enzyme (UBC12), and E3 NEDD8 ligases, which activate and transfer NEDD8 to a target protein successively [9]. Recently, MLN4924, an investigational small-molecule inhibitor of NEDD8-activating enzyme (NAE), has shown antitumor activity in various cancer xenograft models [10-16]. Mechanistically, MLN4924 abrogates cullin neddylation, inactivates CRL, and thus causes accumulation of CRL substrates, which eventually triggers DNA damage, cell cycle arrest, apoptosis and/or senescence in a broad panel of tumor cells [12-19]. Due to its potent anticancer efficacy and well-tolerated toxicity in preclinical studies, MLN4924 is currently tested in several Phase I clinical trials for cancer therapy [20, 21].

In the present study, we investigated the expression of neddylation pathway in human ICC, and tested the preclinical activity of the NEDD8-activating enzyme inhibitor MLN4924 against ICC cells. Using a large consecutive cohort of ICC patients, we found that neddylation pathway was highly active in ICC and possessed independent prognostic value. In established and primary cell lines and xenograft model of ICC, we demonstrated that inhibition of neddylation was a promising strategy for treatment of this malignancy.

## RESULTS

### Neddylation pathway was upregulated in ICC and correlated with clinical outcome

**Figure 1:**
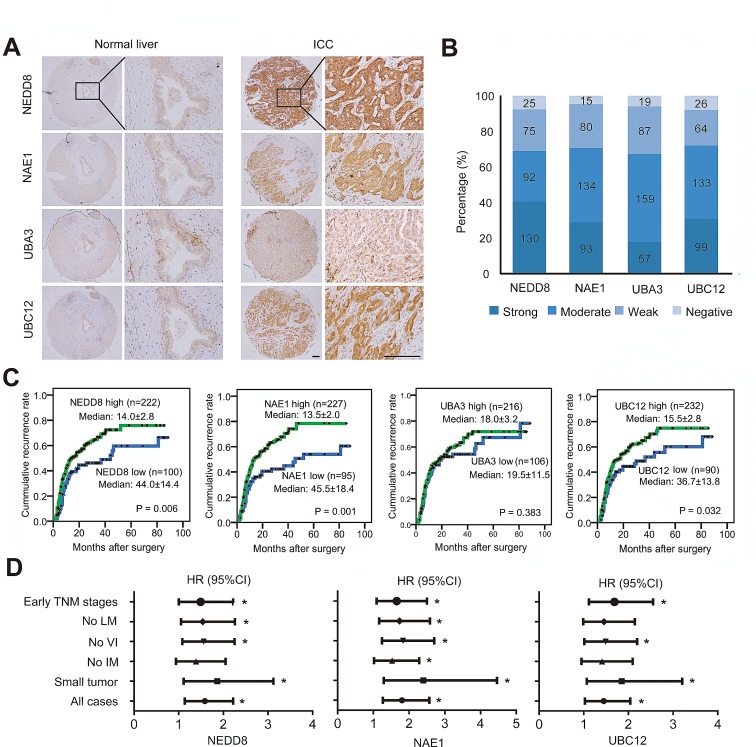
Expression and prognostic significance of neddylation pathway in ICC (A) Representative immunostaining images in ICC tissue arrays using NEDD8, NAE1, UBA3 and UBC12-specific antibodies. Scale bar, 100μm. (B) Bar graph shows the statistics for the staining intensity of NEDD8, NAE1, UBA3 and UBC12 in tissue arrays containing 322 ICC patients. (C) Kaplan–Meier curves for time to recurrence of ICC patients according to the expression of NEDD8, NAE1, UBA3 and UBC12, respectively. (D) Forest plots showing hazard ratio (HR) of time to recurrence for high-risk patients in the indicated clinical subgroups of patients. Univariate Cox analysis was based on the expression of NEDD8, NAE1 and UBC12, respectively. HR >1.0 indicates a worse outcome. *, P < 0.05. CI, confidence interval; LM, lymph node metastasis; VI, vascular invasion; IM, intrahepatic metastasis.

To address the activation status of neddylation pathway in ICC, the expression of E1 (NAE1 and UBA3 subunits) and E2 (UBC12) enzymes as well as global NEDD8 conjugation were determined by immunohistochemical staining in tumor samples from a consecutive cohort of 322 ICC patients. The results showed that, as compared with intrahepatic bile duct cells which showed weak or negative expression of these molecules, high expression of NEDD8 (that represents the global conjugation of NEDD8 to substrates) was observed in 68.9% (222/322; moderate, n=92; strong, n=130) of ICC cases (Fig. 1A-B). Likewise, NAE1, UBA3 and UBC12 were scored as high intensities in 70.5% (227/322; moderate, n=134; strong, n=93), 67.1% (216/322; moderate, n=159; strong, n=57), and 72.0% (232/322; moderate, n=133; strong, n=99) of ICC cases, respectively (Fig. 1A-B). These data indicated that neddylation pathway was upregulated in over two third of human ICC.

To illustrate the clinical relevance of NEDD8, NAE1, UBA3 and UBC12 expression in ICC, patients were dichotomized according to high (strong or moderate intensities) or low (weak or negative intensities) expression of these markers. Univariate analyses revealed that high expression of NEDD8, NAE1, and UBC12 were significantly associated with high risks of postoperative recurrence in ICC patients (Fig. 1C and Supplementary Table S2). The median Time to Recurrence (TTR) for patients with low expression of NEDD8 (44.0 months), NAE1 (45.5 months), and UBC12 (36.7 months) were obviously longer than those with high expression (NEDD8, 14.0 months; NAE1, 13.5 months; UBC12, 15.5 months), respectively (Fig. 1C). Although NEDD8, NAE1, and UBC12 expression significantly correlated with tumor recurrence, their expression intensities were largely independent of conventional clinicopathologic features, like tumor size, vascular invasion, lymph node metastasis, intrahepatic metastasis and TNM stage (Supplementary Table S3). Of note, subgroup analysis showed that the prognostic value of NEDD8, NAE1, and UBC12 also existed in patients with relatively early stage disease, such as patients with small tumor, or with no vascular invasion, or in early TNM stages (I+II) (Fig. 1D).

Furthermore, to further confirm the prognostic significance of NEDD8, NAE1, and UBC12 expression, multivariate Cox proportional hazards regression analysis was performed, adopting all the significant variables in univariate analysis. Multivariate analyses revealed that, among the neddylation pathway components, NAE1 was the only independent factor for postoperative recurrence (P = 0.009) (Supplementary Table S2). Patients with high expression of NAE1 were 1.62 times more likely to suffer from tumor recurrence than those with low expression (HR, 1.62; 95%CI, 1.13-2.33), similar to the predictive power of tumor vascular invasion (HR, 1.65; 95%CI, 1.12-2.43; P = 0.011). However, on multivariate analysis, NEDD8 and UBC12 expression were not associated with TTR any more (Supplementary Table S2). Thus, the results indicated that NAE1 may play a central role in regulating the neddylation pathway and served as an independent prognostic factor for TTR in ICC.

### Relationship among the expression of neddylation pathway components

**Figure 2:**
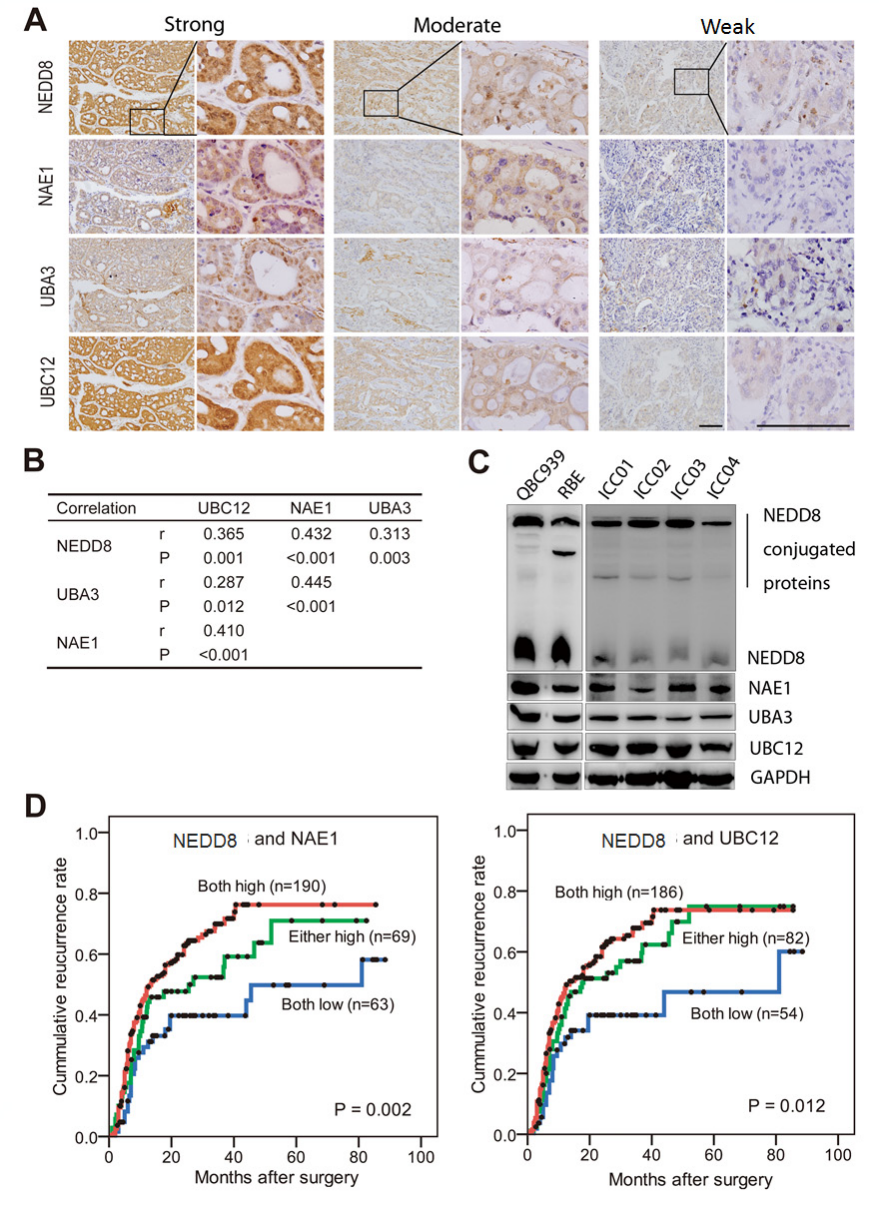
Expression correlation of neddylation pathway components and their combination in recurrence risk prediction (A) Representative immunostaining images showing strong, moderate and weak expression of NEDD8, NAE1, UBA3 and UBC12, respectively. Consecutive slides were stained. Scale bar, 50μm. (B) Statics of expression correlation of neddylation pathway components in 322 ICC patients. (C) Immunobloting analysis of lysates prepared from QBC939 and RBE cells and four primary ICC cell lines. The loading control is GAPDH. (D) Kaplan–Meier curves for time to recurrence applying the combination of NEDD8 and NAE1, or the combination of NEDD8 and UBC12, to stratify patients.

Given that NEDD8, NAE1, UBA3 and UBC12 constituted a highly regulated enzymatic cascade, we explored whether their expression correlated with each other. Notably, using successively-sectioned tumor tissues, significantly positive correlations were found among these components of neddylation pathway (r = 0.287-0.445, P = 0.012 to P < 0.001, Spearman's correlation) (Fig. 2A-B). Immunoblotting showed constitutive expression of the four molecules in established cholangiocarcinoma cell lines (RBE and QBC939) and in-house developed primary ICC cell lines (Fig. 2C). Considering that NAE1 and UBC12 are the NEDD8-activating and -conjugating enzymes respectively, the impact of their combination with NEDD8 on patient prognosis were investigated. Patients with simultaneously high expression of NEDD8 and NAE1 had a strikingly shorter TTR (median, 13.5 months) than patients with NEDD8^low^/NAE1^low^ (median, 81.0 months) (P = 0.002) (Fig. 2D). Likewise, simultaneously high expression of NEDD8 and UBC12 also indicated an obviously reduced TTR (median, 13.5 months), as compared with those with NEDD8^low^/UBC12^low^ (median, 81.0 months) (P = 0.012) (Fig. 2D). Multivariate Cox analysis, adopting conventional clinicopathologic features, confirmed that the combination of NEDD8 and NAE1 was an independent prognostic factor for TTR (Supplementary Table S4). Patients with NEDD8^high^/NAE1^high^ were 2 times more likely to develop recurrence than those with NEDD8^low^/NAE1^low^ (HR, 1.96; 95%CI, 1.25-3.05; P = 0.003).

### MLN4924 inhibited the growth of cholangiocarcinoma cell lines and primary ICC cells

**Figure 3:**
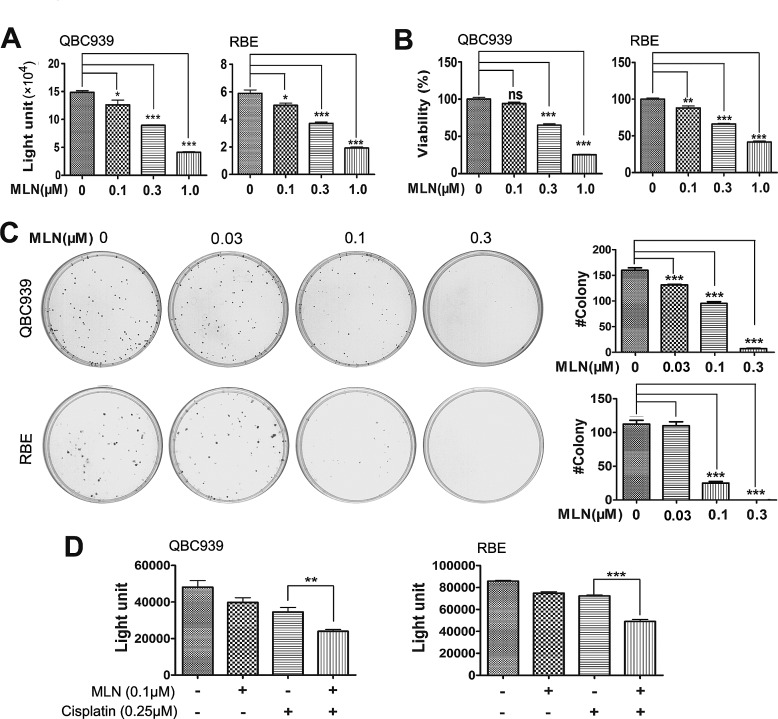
Effects of neddylation inactivation on the growth of cholangiocarcinoma cells (A and B) Effects of MLN4924 on the *in vitro* viability of QBC939 and RBE cells. Cells were treated with the indicated concentrations of MLN4924 for 96 hours and viability was assessed by ATPlite and CCK-8 assays respectively. N = 3 ± SD. (C) Impact of MLN4924 on clonogenic survival. QBC939 and RBE cells were treated with indicated concentrations of MLN4924 for 12 days. N = 3 ± SD. (D) Effects of MLN4924 and cisplatin on the *in vitro* viability of QBC939 and RBE cells. Cells were treated with 0.1μM MLN4924, 0.25 μM cisplatin or both agents for 96 hours. N = 3 ± SD. *, P<0.05; **, P<0.01; ***, P<0.001.

Considering the clinical significance of overactivated neddylation pathway in ICC, we further tested the efficacy of MLN4924, a specific inhibitor of NAE, on cholangiocarcinoma cells QBC939 and RBE. In ATPlite (Fig. 3A) and CCK-8 (Fig. 3B) cell proliferation assays, MLN4924 significantly inhibited the proliferation of cells in a dose-dependent manner. Likewise, MLN4924 caused a dose-dependent inhibition of colony formation of QBC939 and RBE cells (Fig. 3C). In four primary ICC cell lines, MLN4924 effectively suppressed growth of two cell lines with the IC_50_ of 0.378 or 0.629 μM, respectively (Supplementary Fig. S1A-B). However, the other two primary ICC lines showed only moderate sensitivity or resistance to MLN4924, with the IC_50_ of 1.09 or >10 μM, respectively (Supplementary Fig. S1C-D). Thus, these data demonstrated that MLN4924 was a potent inhibitor of cell proliferation and survival in ICC cells. In addition, potential synergy was observed between MLN4924 and cisplatin, an agent used for clinical management of ICC (Fig. 3D), suggesting that MLN4924 favorably impact the anti-tumor activity of cisplatin.

### MLN4924 induced G2 cell-cycle arrest, followed by apoptosis or senescence in cholangiocarcinoma cells

**Figure 4:**
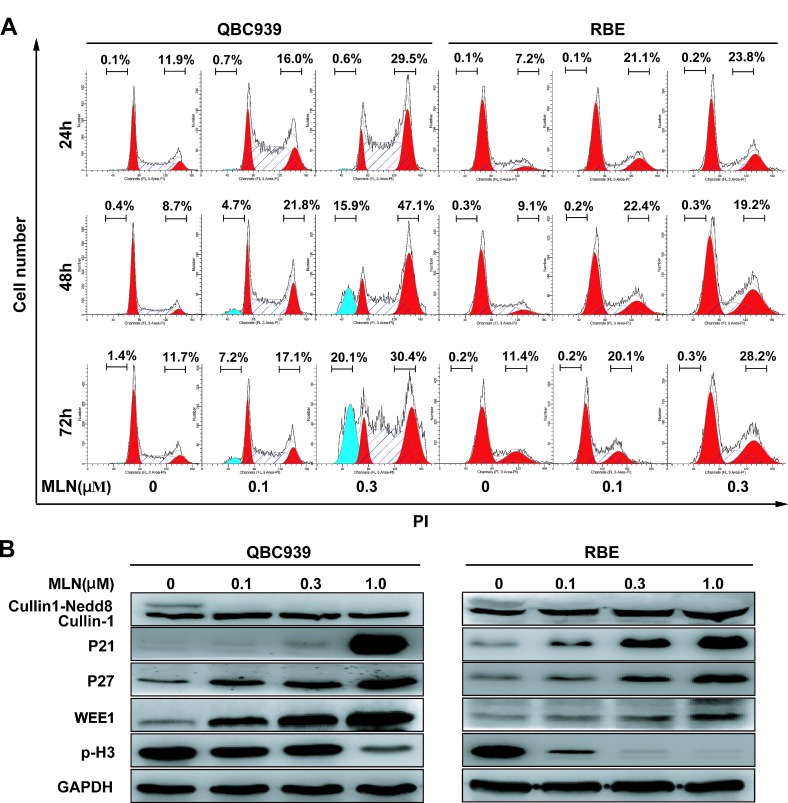
MLN4924 induced G2 cell cycle arrest and apoptosis in cholangiocarcinoma (A) MLN4924 induced cell-cycle arrest in QBC939 and RBE cells, and also apoptosis in QBC939 cells. Cells were treated with 0, 0.1 or 0.3 μM MLN4924, and subjected to PI staining and FACS analysis at indicated time points. The percentages of cells at the G_2_-M phase and sub-G_1_ phase were indicated in figures. (B) MLN4924 induced accumulation of p21, p27, WEE1 and p-Histone H3 (p-H3). Cells were treated with MLN4924 for 72 hours using indicated concentrations and subjected to immunoblotting analysis with GADPH as a loading control.

Triggering cell-cycle disturbance, apoptosis, and senescence were reported to be responsible for anti-tumor effects of MLN4924 [12-19]. Our flow cytometry analysis of DNA content evidenced a prominent increase in G2-M population 24h after treatment in cholangiocarcinoma cells (Fig. 4A). The arrest was confirmed to persist after longer treatment periods (48 and 72 h). In line with the role as a G2-M regulator, sharp increases of cell cycle inhibitors p21, p27, WEE1 (a well defined CRL substrate and an inhibitor of G2-M phase transition) [22], and obvious decrease of a hallmark of M phase, p-Histone H3 (p-H3, ser10) [23], were observed in QBC939 and RBE cells (Fig. 4B). The results indicated that MLN4924-treated cholangiocarcinoma cells were arrested at the G2-phase and failed to enter M-phase.

**Figure 5:**
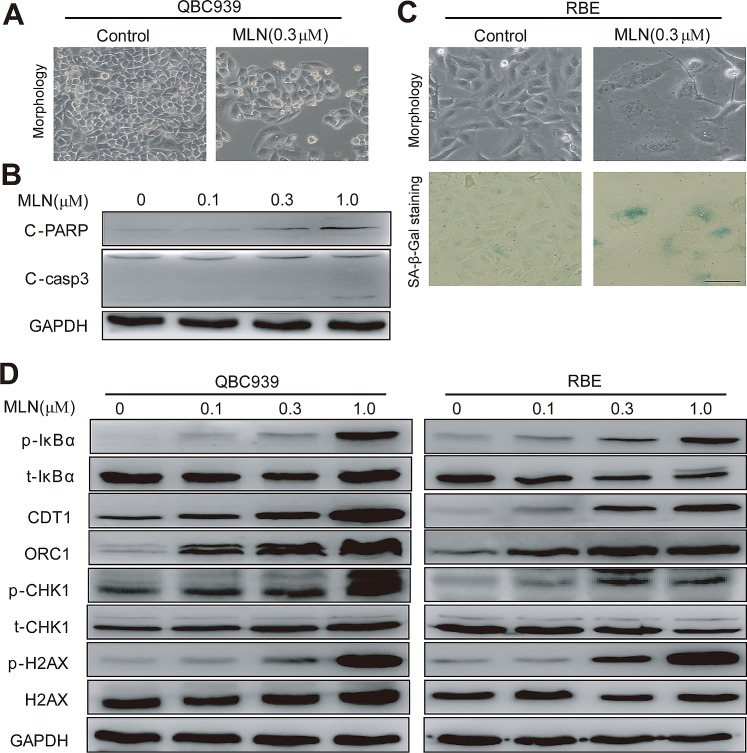
MLN4924 induced apoptosis or senescence in a cell line-dependent manner (A and B) MLN4924 induced apoptosis in QBC939 cells and expression of pro-apoptotic proteins in MLN4924-treated QBC939 cells. Cells were treated with MLN4924 for 72 hours and then subjected to morphological observation, or immunoblotting analysis for proteins involved in apoptotic induction, using GAPDH as a loading control. (C) MLN4924 induced senescence in RBE cells. After 72-hour MLN4924 treatment, cells were subjected to morphological observation and staining of senescence-associated β–galactosidase. Scale bar, 100μm. (D) MLN4924 induced the accumulation of CRL substrates. Cells were treated with MLN4924 for 72 hours using indicated concentrations and subjected to immunoblotting analysis with GADPH as a loading control.

Interestingly, cholangiocarcinoma cells arrested in G2 phase eventually died via either apoptosis or senescence in a cell line-dependent manner. In QBC939 cells, MLN4924 induced apoptosis as reflected by a shrunk morphology in shape (a feature of apoptosis), cleaved caspase 3 and PARP (Fig. 5A-B) and the appearance of sub-G1 peak (Fig. 4A). In contrast, in RBE cells, MLN4924 triggered senescence as demonstrated by an enlarged and flattened cellular shape as well as the expression of senescence-associated β-galactosidase (Fig. 5C).

Further analysis of protein alterations of known CRL substrates was conducted in MLN4924-treated cholangiocarcinoma cells (Fig. 5D). Accumulation of NF-κB inhibitor pIκB-α was observed, which could retain NF-κB subunits in the cytoplasm and decrease their nuclear localization, resulting in reduced cell viability [11, 24]. Meanwhile, accumulation of DNA replication licensing proteins CDT1 and ORC1 were detected, whose over-expression were known to cause double-strand break (DSB) and trigger DNA damage response [17-19], as demonstrated by the appearance of p-H2AX and p-CHK1 (Fig. 5D).

### *In vivo* anti-tumor activity of MLN4924 in cholangiocarcinoma mice models

**Figure 6:**
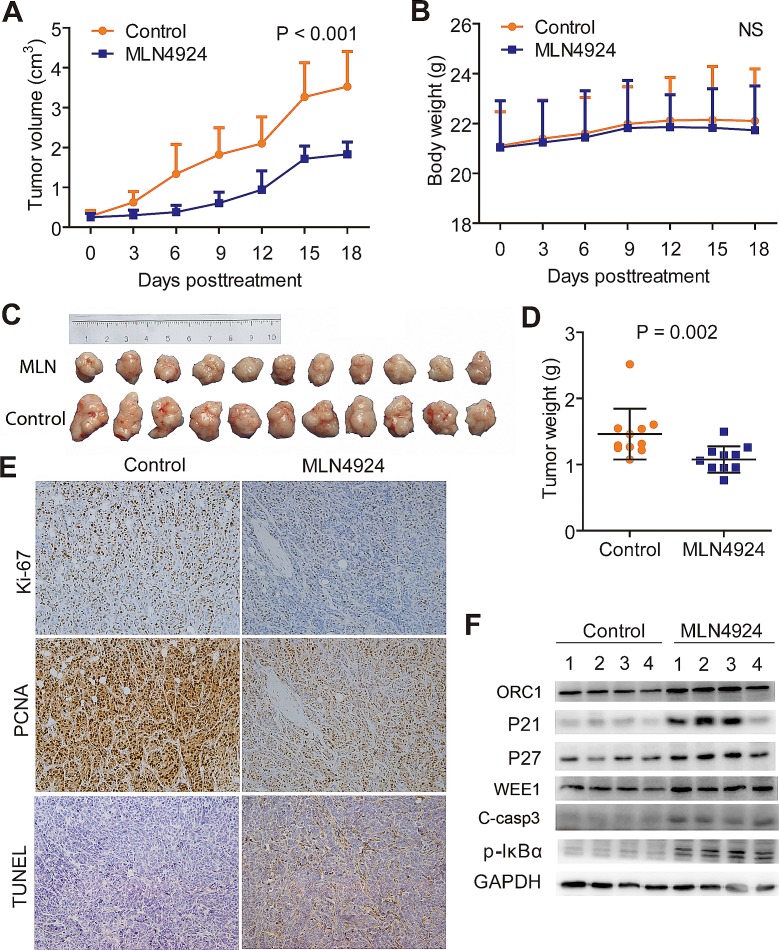
MLN4924 suppressed the growth of subcutaneous xenograft model of cholangiocarcinoma (A) Growth curves showed a significant inhibition on the growth of xenograft tumors under MLN4924 treatment. (B) No obvious toxicity against body weight was observed during MLN4924 treatment. Body weight of mice was measured twice a week during the treatment. (C) Images of MLN4924-treated or control xenograft tumors at the end of experiment (n =11). Tumor tissues of mice were collected, photographed, weighed, and stored for further analyses. (D) MLN4924 significantly reduced tumor weight. (E) Immunostaining showed an obvious decrease in proliferation (Ki-67 and PCNA intensities) and a marked increase in apoptosis (TUNEL) in MLN4924-treated tumors. (F) MLN4924 induced the accumulation of CRL substrates. Four tumor tissues were randomly selected from each group and lysed for immunoblotting analysis as indicated.

To further confirm the anti-tumor effects by targeting NAE1 *in vivo*, MLN4924 was administered to QBC939 subcutaneous xenografts, and the kinetic growth of tumors was monitored. As shown by tumor growth curve (Fig. 6A), MLN4924-treated cholangiocarcinoma grew slowly, whereas control tumors progressed rapidly over time. During the treatment, no obvious treatment-related toxicity against body weight (Fig. 6B), liver function, and kidney function of animals was observed (data not shown). At the end point of MLN4924 treatment, tumors of both treated and control groups were collected, imaged (Fig. 6C), and weighed (Fig. 6D). As shown in Fig. 6C, the size of MLN4924-treated cholangiocarcinoma was significantly smaller than that of control tumors. Consistently, the weight of MLN4924-treated cholangiocarcinoma was significantly lighter than that of control tumors (Fig. 6D). The results clearly indicated that MLN4924 had a strong anti-tumor activity against cholangiocarcinoma *in vivo* and was well tolerated in mice.

To explore the *in vivo* mechanisms of MLN4924, immunohistochemical analysis of proliferative and apoptotic index were conducted. The results showed a marked decrease of PCNA and Ki-67 intensities indicating the suppression of cell proliferation, and an obvious increase in TUNEL staining indicating the induction of apoptosis in MLN4924 treated tumors, as compared with control tumors (Fig. 6E). Finally, tissue samples from four tumors each group were randomly selected to determine the inactivation status of CRL by measuring the expression of classical tumor-suppressive CRL substrates. Consistent with *in vitro* experiments, sharp increases of ORC1, p21, p27, WEE1 and C-casp3, as well as accumulation of pIκB-α, were observed in MLN4924-treated tumors (Fig. 6F).

## DISCUSSION

Herein, for the first time, we revealed the clinical significance of neddylation pathway overactivation and reported preclinical evaluation of inhibiting this pathway using MLN4924, a potent and selective small-molecule inhibitor of NEDD8-activating enzyme [10], in ICC. We found that overactivation or up-regulation of neddylation pathway occurred in about two third of ICC patients, and high expression of certain components of neddylation pathway significantly and independently correlated with increased tumor recurrence. Moreover, we demonstrated that MLN4924 had a broad tumoricidal activity both *in vivo* and *in vitro*. Similarly, we and others recently had reported that neddylation pathway was overactivated in several types of human cancer and inhibition of this pathway significantly inhibited tumor growth [12-19]. Thus, these data provided additional information on the crucial role of neddylation pathway in human cancers and, in particular, a rationale for targeting this pathway in patients with ICC.

The therapeutic potential of modulating components of the UPS and UBL conjugation pathways in cancer has been demonstrated by the proteasome inhibitor bortezomib (Velcade; Millennium Pharmaceuticals) [4, 25]. Further efforts to search other druggable targets in these pathways had identified NEDD8-activating enzyme as the focus of particular interest, which could catalyze the first step in the neddylation pathway [20, 26]. In this study, our interest in targeting NEDD8-activating enzyme for treatment of ICC was further stimulated by the observation that expression of neddylation pathway components, like NEDD8, NAE1, UBA3 and UBC12, was up-regulated in more than two third of human ICC and significantly positive correlations were found among them. More importantly, NAE1 was identified as an independent factor for postoperative recurrence and a combination of NAE1 and NEDD8 provided a better prediction for patient prognosis. Similar results have also been reported in human lung cancer and colon cancer, where expression of neddylation pathway correlated with cancer progression and poor prognosis [16, 27].

Then, we demonstrated significant anti-tumor and chemosensitizing/synergizing effects of MLN4924 both in established and primary cholangiocarcinoma cell lines, as well as in mice xenograft model with low toxicity. Interestingly, MLN4924 induced cell death via either apoptosis or cellular senescence in a cell line-dependent manner. Mechanistically, the anticancer efficacy of MLN4924 is mainly attributed to the inhibition of CRL activity and the accumulation of CRL substrates. For examples, MLN4924 treatment led to the accumulation of (a) G2-M phase transition inhibitor WEE1 that led to G2 phase cell cycle arrest [10, 14], (b) DNA replication licensing protein CDT1 and ORC1 that triggered DNA re-replication stress and DNA damage [10, 14, 17-19], (c) tumor suppressor p21 and p27 that resulted in cell senescence [19], and (d) pro-apoptotic proteins such as NOXA that induced apoptosis [16]. Among these substrates, NOXA was exclusively induced in some of but not all treated cells, which led to cell-context induction of apoptosis [16]. Further studies are required to address the mechanisms for cell-line dependent induction of pro-apoptotic proteins (e.g. NOXA) which plays a critical role in cell fate determination (apoptosis vs senescence) upon neddylation disruption with MLN4924. In addition, dramatic accumulation of IκB-α was observed in treated cells. Considering that induction of NF-κB signaling was an important mechanism of resistance to many chemotherapeutic and targeted agents [28, 29], MLN4924 may be helpful to overcome NF-κB-related treatment resistance.

The anti-tumor potential of MLN4924 in ICC was largely in line with those findings in hematological malignancies and other solid tumors, supporting the rationale to develop phase I trials for evaluating MLN4924 in ICC. However, one of the primary ICC cell line with the lowest expression of NAE1 were highly resistant to MLN4924 treatment, indicating the presence of primary resistance to MLN4924 in ICC, and highlighting the need for precise patient selection according to the activation status of neddylation pathway. Similarly, treatment-induced mutations of NAEβ/UBA3 were reported to lead to secondary resistance to MLN4924 in leukemia [30, 31], further mandating the development of combinational regiments including MLN4924 or novel next-generation NAE inhibitors.

In summary, our study demonstrated that neddylation pathway was upregulated/overactivated in ICC and served as a promising therapeutic target, which provided impetus for clinical trials of MLN4924 in the treatment of ICC. Due to the primary resistance to MLN4924, expression status of neddylation pathway may provide a base for appropriate patient enrollment, although further validation was need. Meanwhile, discovery of new neddylation inhibitors, such as inhibitors of neddylation E2 or E3 and next-generation E1 inhibitors may provide additional choice for targeting overactivated neddylation pathway and overcome the primary/secondary resistance.

## MATERIAL AND METHODS

### Patients and specimens

Paraffin-embedded tissue samples from 322 consecutive ICC patients who underwent primary and potentially curative resection for their tumor in Liver Cancer Institute, Zhongshan Hospital of Fudan University (Shanghai, China) between 2005 and 2011 were selected. Patients had no signs of distant metastasis nor had any anticancer treatments before surgery. The clinical information of these patients is provided Supplementary Table S1 and also in our previous study [32].

The patient follow-up and postoperative management were administrated abiding our established guidelines as described previously [32, 33]. The median duration of follow-up of the 322 patients was 21.0 months (range, 3.0-91.5 months; SD, 18.3 months). TTR was defined as the interval between the date of surgery and the first recurrence, or from the date of surgery to the date of last follow-up for the patients without recurrence. Data were censored at last follow-up for patients without relapse. The study was approved by the Zhongshan Hospital Ethics Committee, and informed consent was obtained from each patient under Institutional Review Board protocols.

### Tissue microarray and immunohistochemistry

Tissue microarrays were constructed as previously described [32, 33]. Core samples were obtained from representative regions from each tumor on hematoxylin and eosin staining. Duplicate 1-mm cores were taken from different areas of the same tissue block for each case (tumor tissue and matched noncancerous liver tissue, i.e., a total of four cores). Tissue microarrays were constructed using an arraying machine (Beecher Instruments).

Immunohistochemistry was performed as previously described. Briefly, 4-μm sections were deparaffinized and subjected to antigen retrieval (citrate buffer, pH=6.0). Sections were then incubated for 30 min with goat polyclonal antibody to NEDD8, NAE1, UBA3 and UBC12 (Santa Cruz Biotechnology). Reaction products were visualized with 3, 3'-diaminobenzidine tetrahydrochloride and counterstained with hematoxylin. NEDD8, NAE1, UBA3 and UBC12 immunostaining intensities were semiquantitatively scored as: 0, negative; 1, weak; 2, moderate; 3, strong by two observers independently, and comparisons were made between tumor/normal pairs. In subsequent analyses, sores 2 and 3 were defined as high expression, while scores 0 and 1 indicated low expression of each molecules.

### Cell lines and agents

Human cholangiocarcinoma cell lines QBC939 (donated by Professor Wang SG at the Third Military Medical University, China) and RBE (Cell Resource Center of Tohoku University, Japan) were cultured in Dulbecco's Modified Eagle's Medium (Hyclone), containing 10% FBS (Biochrom AG) and 1% penicillin–streptomycin solution, at 37°C with 5% CO_2_. MLN4924 was synthesized as previously described (34). For *in vitro* studies, MLN4924 stock solution (10 mM) was prepared in dimethyl sulfoxide (DMSO) and stored at -20°C as small aliquots until needed. For *in vivo* studies, MLN4924 was dissolved in 10% 2-hydroxypropyl-b-cyclodextrin (HPBCD), and the solution of MLN4924 was freshly made every week and stored in dark at room temperature before use [10, 14].

Four primary ICC cell lines were established using freshly resected human ICC samples, essentially as previous described [35]. Each line was validated by its unique DNA short tandem repeat “fingerprints” matching that of the patient's tumor tissue.

### Cell viability assessment and IC50 determination

QBC939 and RBE cells seeded in 96-well plates with 1500 cells per well in triplicate were treated with MLN4924 or DMSO for 96 hours. Cell viability was determined using the ATPlite^TM^ kit (PerkinElmer) and CCK8 (Dojindo), following the manufacturer's instructions and as previously described [14, 16].

For IC_50_ determination, primary ICC cells were seeded with 2000-4000 cells per well in 96-well plates according to the nature of each lines. MLN4924 was given from 5nM to 50μM serially. Cell viability on day 7 was determined using the ATPlite^TM^ kit (PerkinElmer) following the manufacturer's instructions.

### Clonogenic assay

For clonogenic assay, cells were seeded into 6-well plates (200 cells per well) and cultured for 12 days. The colonies on the plate were fixed with 4% paraformaldehyde and stained with crystal violet. The colonies with more than 50 cells were counted.

### Propidium iodide staining and fluorescence activated cell sorting analysis

Cells treated with MLN4924 or DMSO were harvested and fixed in 70% ethanol at -20°C overnight, and stained with propidium iodide (PI, 36 μg/ml, Sigma) containing RNAase (10 μg/ml, Sigma) at 37°C for 15 min, then analyzed for apoptosis and cell cycle profile by CyAnTM ADP (Beckman Coulter) as previously described [14, 16]. Data were analyzed with ModFit LT software. Apoptosis was measured by the percentage of cells in sub-G1 population.

### SA-β-Galactosidase Staining

The expression of senescence-associated β-galactosidase was determined by SA-β-Galactosidase (SA-β-Gal) staining (Beyotime) according to the manufacturer's instructions and as previously described [36, 37].

### Immunoblotting analysis

Immunoblotting was performed as previously described [14,16]. Cell lysates were prepared for immunoblotting, using antibodies against NEDD8, NAE1, UBA3, UBC12, WEE1, cullin-1(Santa Cruz Biotechnology), ORC1, p27, cleaved Caspase 3, cleaved PARP, CDT1, H2AX, p-H2AX, CHK1, p-CHK1, GAPDH, p-Histone H3 (Cell Signaling), IκB-α, p-IκB-α, p21 (Epitomics, Inc.).

### Animal experiments

Five-week-old male athymic nude mice were obtained from the Shanghai experimental animal center (Shanghai, China). QBC939 cells were trypsinized, resuspended in PBS and then subcutaneously injected into groin with 5×10^6^ cells per injection. Three days later, the tumor bearing mice were randomly divided into 2 groups (11 mice/group), started to treat with 10% HPBCD or MLN4924 (60 mg/kg, s.c.), twice a day respectively, on a 5-days-on/2-days-off schedule for 5 cycles within total 25 days. The size of tumors was measured by Vernier caliper twice a week. At the end of study, mice were sacrificed, and tumor tissues were collected, photographed and weighted. Fresh frozen tumor tissues were used for immunoblotting assay, and paraffin-embed tissues were sectioned for immunohistochemistry of Ki-67 and PCNA (Santa Cruz Biotechnology) or TUNEL assay (Promega). All procedures were performed in accordance with the National Institutes of HealthGuide for the Care and Use of Laboratory Animals.

### Statistical analysis

Statistical analyses were performed with SPSS version 18.0 for Windows (IBM). Data were presented as the mean ± SD. Fisher's exact, spearman's correlation coefficients, and Mann-Whitney *U* test were used where appropriate. The relationship between the TTR and prognostic variables was analyzed using Kaplan-Meier methods (log-rank test). Univariate and multivariate analyses were based on the Cox proportional hazard regression model. P < 0.05 was considered statistically significant.

### SUPPLEMENTARY MATERIAL FIGURE AND TABLES


