# Supplementary material for: Neddylation pathway is up-regulated in human intrahepatic cholangiocarcinoma and serves as a potential therapeutic target
Source: Oncotarget. 2014 Aug 4;5(17):7820–32. doi: 10.18632/oncotarget.2309 (PMC4202163; doi:10.18632/oncotarget.2309)
Supplement: Supplementary file 1 [file oncotarget-05-7820-s001.pdf]

Neddylation pathway is up-regulated in human intrahepatic cholangiocarcinoma and serves as a potential therapeutic target

Supplementary Material

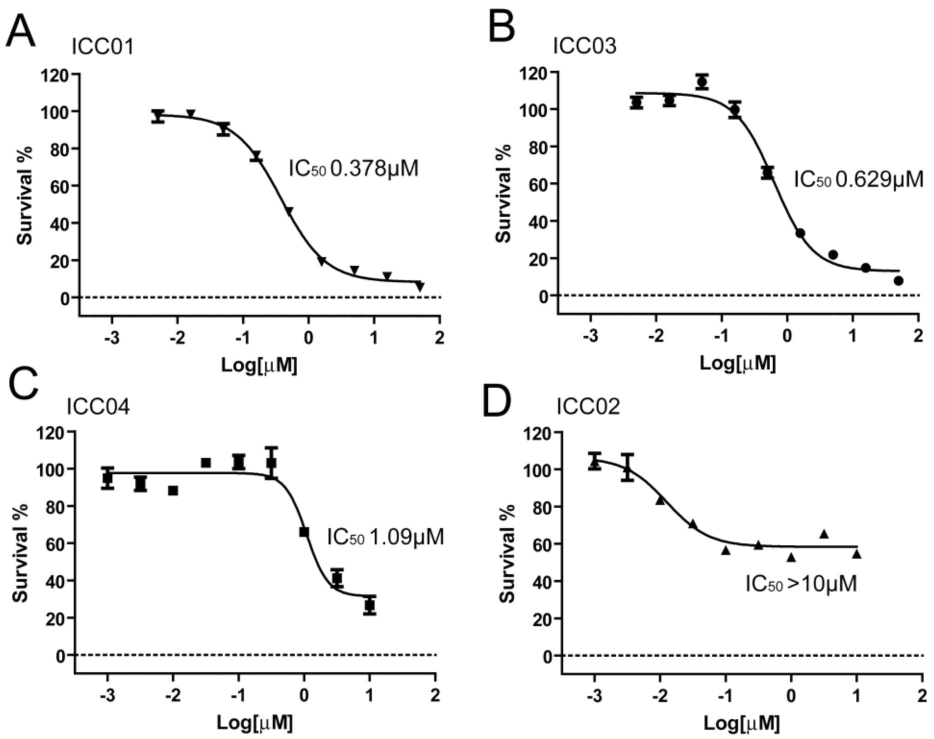

Supplementary Figure S1: Efficacy of growth suppression of primary ICC cells by MLN4924. Primary ICC cell lines were established as described in the Methods. Cells were seeded in 96-well plates in triplicates and treated with various concentrations of MLN4924for 7 days, respectively. Cells were then lysed for ATPlite assay. N = 3 ± SD.

Supplementary Table S1: Demographics and Clinicopathologic Features of Patients with Intrahepatic Cholangiocarcinoma

**Variables**	**No.**	**%**
Age: median (IQR), years	58.0	50.0-65.3
Male gender:	194	60.2
CA19-9: median (IQR), U/ml	37.4	14.7-231.6
Etiology:		
HBV	124	38.5
lithiasis	8	2.5
liver fluke	11	3.4
unknown	179	55.6
Liver cirrhosis	86	26.7
Tumor size: median (IQR), cm	6.0	4.0-8.0
Tumor differentiation[a]:		
well	16.5	16.5
moderate	62.4	62.4
poor	21.1	21.1
Lymph node metastasis	56	17.4
Vascular invasion	46	14.3
Intrahepatic metastasis	78	24.2
TNM stage[b]:		
I	167	51.9
II	81	25.2
III	16	5.0
IVA	58	18.0
Tumor recurrence	184	57.1

Abbreviations: HBV, hepatitis B virus; TNM, tumor–node–metastasis; IQR,

interquartile range.

[a] Tumor differentiation was determined according to the "British Society of Gastroenterology guidelines on the management of cholangiocarcinoma".

[b] American Joint Committee on Cancer 7th edition staging for intrahepatic cholangiocarcinoma.

Supplementary Table S2. Univariable and Multivariable Analyses of Factors Associated with Time to Recurrence in 322 Intrahepatic Cholangiocarcinoma Patients

**Variables**	**Univariable [a]**		**Multivariable [b]**	
	**Hazard ratio (95% CI)**	***P***	**Hazard ratio (95% CI)**	***P***
Age: >58 v ≤58 (year)	0.91 (0.68-1.22)	.520		NA
Gender: male v female	1.05 (0.78-1.42)	.737		NA
CA19-9: >37 v ≤ 37 (U/ml)	1.37 (1.01-1.85)	.041		NS
Liver cirrhosis: yes v no	1.25 (0.91-1.73)	.170		NA
Tumor size: >5 v ≤5 (cm)	1.33 (0.99-1.78)	.059		NA
Tumor differentiation: poor v moderate v well	0.99 (0.78-1.26)	.955		NA
Lymph node metastasis: yes v no	2.56 (1.81-3.63)	<.001	2.47 (1.72-3.55)	<.001
Vascular invasion: yes v no	1.59 (1.10-2.31)	.015	1.65 (1.12-2.43)	.011

Intrahepatic metastasis: yes v no	1.80 (1.31-2.47)	<.001	1.74 (1.25-2.43)	.001
TNM stage: IVA+III v II+I	2.04 (1.47-2.81)	<.001		NS
NEDD8: high v low	1.59 (1.14-2.22)	.007		NS
NAE1: high v low	1.80 (1.26-2.57)	.001	1.62 (1.13-2.33)	.009
UBA3: high v low	1.15 (0.84-1.57)	.388		NA
UBC12: high v low	1.45 (1.03-2.04)	.034		NS

Abbreviations: TNM, tumor–node–metastasis; CI, confidence interval; NA, not applicable; NS, not significant.

[a] Cox proportional hazards regression.

[b] Cox proportional hazards regression analysis adjusting for CA19-9 level, lymph node metastasis, vascular invasion, intrahepatic metastasis and TNM stage, using stepwise selection.

Supplementary Table S3. Relationship of NEDD8, NAE1, UBA3 and UBC12 Expression with Clinicopathologic Features of ICC.

**Variables**	**NEDD8**			**NAE1**			**UBA3**			**UBC12**		
	**low**	**high**	***P***	**low**	**high**	***P***	**low**	**high**	***P***	**low**	**high**	***P***
Age												
≤58 year	48	121	0.335	45	124	0.271	48	121	0.076	49	120	0.710
>58 year	52	101		50	103		58	95		41	112	
Gender												
female	44	84	0.326	45	83	0.081	53	75	**0.011**	39	89	0.447
male	56	138		50	144		53	141		51	143	
Liver cirrhosis												

no	77	159	0.343	74	162	0.270	79	157	0.789	66	170	1.00
yes	23	63		21	65		27	59		24	62	
Tumor size												
≤5 cm	47	98	0.717	38	107	0.270	49	96	0.812	41	104	1.00
>5 cm	53	124		57	120		57	120		49	128	
Tumor differentiation												
well	20	33	0.181	20	33	0.107	24	29	0.049	19	34	0.063
moderate	55	146		51	150		57	144		47	154	
poor	25	43		24	44		25	43		24	44	
Lymph node metastasis												
no	83	183	1.00	80	186	0.747	88	178	1.00	76	190	0.627
yes	17	39		15	41		18	38		14	42	
Vascular invasion												
no	87	189	0.733	83	193	0.727	96	180	0.091	74	202	0.288
yes	13	33		12	34		10	36		16	30	
Intrahepatic metastasis												
no	79	165	0.401	76	168	0.318	86	158	0.129	72	172	0.312
yes	21	57		19	59		20	58		18	60	
TNM stage [b]												
I+ II	74	174	0.394	72	176	0.772	80	168	0.673	70	178	0.884
III+ IVA	26	48		23	51		26	48		20	54	
Recurrence												
no	54	84	**0.008**	54	84	**0.001**	50	88	0.283	47	91	**0.044**
yes	46	138		41	143		56	128		43	141	

Fisher’s exact tests or Chi-square tests.

Supplementary Table S4: Multivariable Analyses of Factors Associated with Time to Recurrence in 322 Intrahepatic Cholangiocarcinoma Patients

Variables	Multivariable [a]	
	Hazard ratio (95% CI)	*P*
CA19-9: >37 v ≤ 37 (U/ml)		NS
Lymph node metastasis: yes v no	2.52 (1.76-3.63)	<.001
Vascular invasion: yes v no	1.69 (1.15-2.50)	.008
Intrahepatic metastasis: yes v no	1.75 (1.25-2.44)	.001
TNM stage: IVA+III v II+I		NS
NEDD8 and NAE1		
Overall		.010
Either high v both low	1.52 (0.90-2.56)	.117
Both high v both low	1.96 (1.25-3.05)	.003
NEDD8 and UBC12		NS

Abbreviations: TNM, tumor–node–metastasis; CI, confidence interval; NS, not significant.

[a] Cox proportional hazards regression analysis adjusting for CA19-9 level, lymph node metastasis, vascular invasion, intrahepatic metastasis and TNM stage, using stepwise selection.
